# A Sensitive Sandwich-Type Electrochemical Immunosensor for Carbohydrate Antigen 19-9 Based on Covalent Organic Frameworks

**DOI:** 10.3390/bios15080492

**Published:** 2025-08-01

**Authors:** Ting Wu, Rongfang Chen, Yaqin Duan, Longfei Miao, Yongmei Zhu, Li Wang

**Affiliations:** 1 College of Chemistry and Materials, Jiangxi Normal University, Nanchang 330022, China; wuting@jxnu.edu.cn (T.W.);; 2 School of New Energy Science and Engineering, Xinyu University, Xinyu 338004, China

**Keywords:** covalent organic frameworks, carbohydrate antigen 19-9, signal amplification strategy, electrochemical immunosensor

## Abstract

Since carbohydrate antigen 19-9 (CA 19-9) is a significant biomarker for the clinical diagnosis and treatment of pancreatic cancer, a sensitive sandwich-type immunosensor was proposed with an epoxy functionalized covalent organic framework (EP-COF_TTA-DHTA_) as the antibody carrier and an electroactive COF_TTA-2,6-NA(OH)2_ as the signal amplification probe for the sensitive detection of CA 19-9. The flexible covalent linkage between the epoxy-functionalized EP-COF_TTA-DHTA_ and the antibodies was employed to improve the dynamics of the antigen–antibody interaction significantly. Meanwhile, AuNPs@COF_TTA-2,6-NA(OH)2_ with abundant electroactive sites enhanced the current response of the immunoreaction significantly. After optimizing the incubation time and concentration of the antibody, CA 19-9 was quantitatively detected by differential pulse voltammetry (DPV) based on the sensitive sandwich-type immunosensor with a low detection limit of 0.0003 U/mL and a wide linear range of 0.0009–100 U/mL. The electrochemical immunosensor exhibits high specificity, stability and repeatability, and it provides a feasible and efficient method for the pathologic analysis and treatment of tumor markers.

## 1. Introduction

Pancreatic cancer is a digestive system tumor with high mortality, and early diagnosis is very important for tumor monitoring and treatment [1,2,3]. Carbohydrate antigen 19-9 (CA 19-9) is a potential biomarker of pancreatic cancer owing to its higher specificity and sensitivity for pancreatic cancer [4,5,6]. Various methods have been devised to identify CA 19-9 [7,8,9,10,11], including surface-enhanced Raman scattering spectroscopy (SERS) [8], chemiluminescent immunoassay (CL) [9], enzyme-linked immunosorbent assay (ELISA) [10] and radioimmunoassay (RIA) [11]. However, complex and time-consuming procedures, expensive instruments and high technical requirements have seriously limited the widespread application of these technologies. An electrochemical immunosensor combines the specificity of the immunoreaction with the simplicity and low cost of the electrochemical sensor, which makes it present a great prospect for point-of-care detection of CA 19-9 [12,13,14]. However, there are still significant obstacles to developing ultrasensitive electrochemical immunosensors.

Various efforts have been carried out to enhance the sensitivity. Porous materials such as mesoporous silica were applied in constructing markers or supporters to enrich the signal sources [15]. Dendrimers, as monodisperse and repeating branched chain macromolecules, have also been used to enrich the signal sources for the design of excellent response labels [16]. DNA amplification strategies for the enrichment of redox species were also developed [17,18]. The controllable assembly of the nanometer-sized complexes by means of host–guest chemical reactions has been used widely to improve the sensitivity of electrochemical immunosensors [19,20]. The signal amplification strategy is another effective way to improve the sensitivity of electrochemical immunosensing [21]. It is worth considering that the mass transfer efficiency and conductivity of the electrode carrier materials can play a key role in the signal response during the immunoassay process. So, the development of carrier materials with excellent characteristics and optimization of their structure and performance have led to a significant improvement through improving the bearing capacity of the signal species. In a word, it is vital to design an efficient signal amplification strategy and identify suited functional materials to improve the sensitivity of electrochemical immunosensors.

Covalent organic framework (COF) is a kind of porous crystalline framework that is composed of light elements (C, O, N, B, etc.) linked with covalent bonds, and it usually presents two-dimensional (2D) nanosheets [22,23,24]. COFs exhibit some great advantages, including an ultra-large specific surface area, controllable pore size/structure, good chemical stability, etc. [25,26,27,28,29]. The application of COFs as carrier materials to construct immunosensors is a wise approach [30,31]. Its periodic structure can load large amounts of electroactive substances, which greatly amplify the current response of the electrochemical immunosensor [32,33,34]. The function of COFs can easily be designed by selecting the appropriate monomer or post-modification, which will facilitate the construction of COF-based immunosensor probes, and the well-ordered controllable pore facilitates mass transfer. The 2D COF nanosheets will ensure that the electroactive material loaded on the COFs can transfer electrons efficiently between the electrode and the loaded electroactive material after forming a sandwich structure. In addition, COFs have good biocompatibility, which is conducive to anchoring antibody biomolecules firmly [35,36]. So, COFs have been used to prepare electrochemical immunosensors. For example, the use of electroactive COF_DAAQ-TFP_ as a beacon to design a sandwich-type carcinoembryonic antigen electrochemical immunosensor [37]. Based on the signal amplification strategy of electropositive COFs, the immunosensor of Cancer Antigen 125 (CA125) was developed [38]. The NSE electrochemical immunosensor, through catalysis of ascorbic acid by heteropolysate COFs, was also prepared [39]. Previous works have proved that a COF is an excellent material for preparing a sandwich-type electrochemical immunosensor.

In this work, a novel sandwich-type electrochemical immunosensor was proposed based on two kinds of precision-regulated COFs for ultrasensitive detection of CA 19-9. Specifically, abundant epoxy functional groups were modified on the COF_DHTA-TTA_ surface, and then Ab_1_ was stably loaded onto the EP-COF_TTA-DHTA_ surface through covalent bonding between the epoxy functional groups and the amino of Ab_1_. Then, Au nanoparticles (AuNPs) were modified on the surface of electroactive COF_TTA-2,6-NA(OH)2_ for the loading of Ab_2_ via the Au–S bond and enhancement of the electrochemical signals simultaneously. The abundant electroactive sites of COF_TTA-2,6-NA(OH)2_ facilitated the enrichment of the signal probes effectively, which realized ultra-sensitive and reliable detection of trace amounts of CA 19-9.

## 2. Materials and Methods

### 2.1. Synthesis of COF_TTA-DHTA_ and COF_TTA-2,6-NA(OH)2_

COF_TTA-DHTA_ and COF_TTA-2,6-NA(OH)2_ were prepared by the amine–aldehyde condensation reaction. The detailed procedures and the chemicals and instruments are presented in the Supplementary Materials (Figures S1 and S2).

### 2.2. Synthesis of EP-COF_TTA-DHTA_

EP-COF_TTA-DHTA_ was prepared by Williamson ether reactions between COF_TTA-DHTA_ and epibromohydrin (Figure S3) [40]. Typically, the COF_TTA-DHTA_ (30 mg), epibromohydrin (25 μL, 0.3 mmol) and potassium carbonate (41.5 mg, 0.3 mmol) were added into degassed anhydrous DMF (5 mL) under an N_2_ atmosphere. After stirring at 120 °C for 1 day, the product was centrifugally cleaned with DMF, methanol and n-hexane, respectively. Finally, the product was dried at 60 °C for 12 h to give EP-COF_TTA-DHTA_.

### 2.3. Construction of Ab_2_/AuNPs@COF_TTA-2,6-NA(OH)2_ Signal Probe

First, 20 mg of COF_TTA-2,6-NA(OH)2_ was added into 20 mL of ultrapure water and ultrasonically dispersed for half an hour. Then, 1 mL of 15 mg/mL HAuCl_4_ was added quickly and stirred vigorously for 1 h. Next, 4 mL of 10 mg/mL trisodium citrate dihydrate was added and stirred for 12 h. The product was centrifuged three times with ultrapure water and dried in an oven at 60 °C to get AuNPs@COF_TTA-2,6-NA(OH)2_. Then, 10 mg of AuNPs@COF_TTA-2,6-NA(OH)2_ was added to 1 mL of 0.2 M PBS (pH 7.0), ultrasonically dispersed for 10 min, and 2 mL of 80 μg/mL Ab_2_ was further added and incubated overnight at 4 °C. Next, 1 mL of 1% BSA was added and incubated for 2 h. Finally, the product was centrifuged three times with PBS (pH 7.0) and dissolved in 1 mL of 0.2 M PBS to obtain a beacon of Ab_2_/AuNPs@ COF_TTA-2,6-NA(OH)2_.

### 2.4. Detection of CA 19-9

**Scheme 1:**
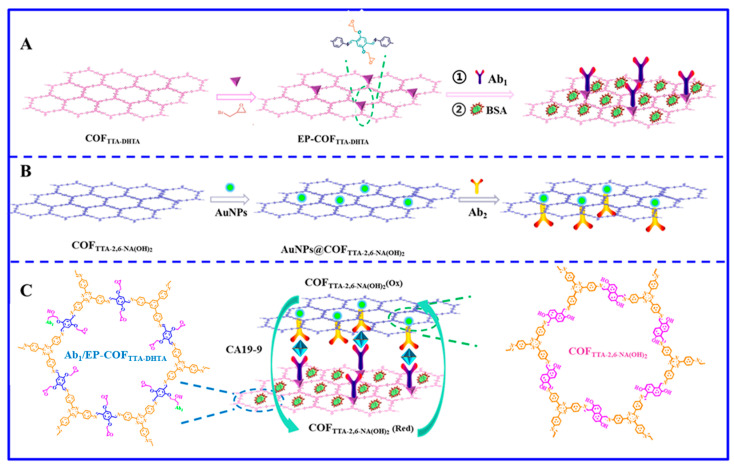
Schematic illustration of the electrochemical immunosensor. (**A**): Construction of the base part of the immunosensor; (**B**): Construction of Ab_2_/AuNPs@COF_TTA-2,6-NA(OH)2_ Signal Probe; (**C**): The mechanism of the immunosensor.

First, 5 μL of 2 mg/mL EP-COF_TTA-DHTA_ was dropped onto the polished GCE to give EP-COF_TTA-DHTA_/GCE. Next, EP-COF_TTA-DHTA_/GCE was incubated in 80 μg/mL Ab_1_ for 4 h, and the EP-COF_TTA-DHTA_ was linked to the Ab_1_ by an epoxy functional group covalently binding to the amino group of the antibody (Figure S4) [37]. Then, the Ab_1_/EP-COF_TTA-DHTA_/GCE was exposed to 1 mL of 1% BSA to hinder some nonspecific binding sites. Subsequently, Ab_1_/EP-COF_TTA-DHTA_/GCE was placed in CA 19-9 solution at 37 °C for 1 h and rinsed thoroughly with 0.2 M PBS (pH 7.0) to remove those loosely bound molecules. Finally, CA 19-9/Ab_1_/EP-COF_TTA-DHTA_/GCE was soaked in 2 mg/mL beacon solution of Ab_2_/AuNPs@COF_TTA-2,6-NA(OH)2_ for 40 min to obtain AuNPs@COF_TTA-2,6-NA(OH)2_/Ab_2_/CA 19-9/Ab_1_/EP-COF_TTA-DHTA_/GCE. The whole preparation is illustrated in Scheme 1.

## 3. Results and Discussion

### 3.1. Characterization of Ab_1_/EP-COF_TTA-DHTA_

**Figure 1:**
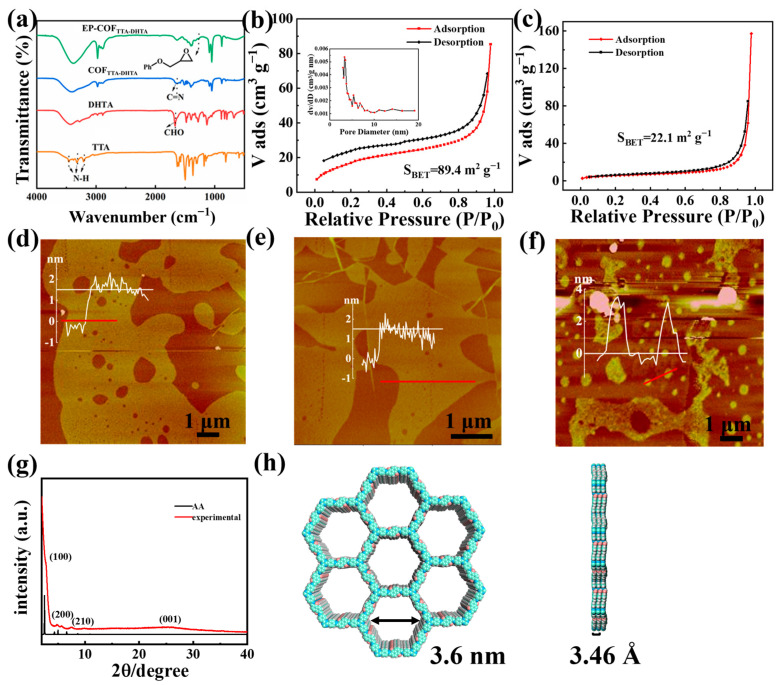
The –OH of COF_TTA-DHTA_ was successfully replaced by the epoxy functional group from the FTIR spectra of TTA (orange), DHTA (red), COF_TTA-DHTA_ (blue) and EP-COF_TTA-DHTA_ (green) (**a**). The N_2_ adsorption and desorption isotherm of COF_TTA-DHTA_ (**b**), the illustration is the aperture distribution of COF_TTA-DHTA_) and EP-COF_TTA-DHTA_ (**c**), where the epoxy functional groups have significantly reduced the BET value of EP-COF_TTA-DHTA_. AFM images of COF_TTA-DHTA_ (**d**), EP-COF_TTA-DHTA_ (**e**) and AFM of Ab_1_/EP-COF_TTA-DHTA_ (**f**) (red line is the cross-sectional height). Experimental (red) and refined (black) XRD patterns of the COF_TTA-DHTA_ (**g**). Eclipsed conformations of COF_TTA-DHTA_ and the π–π stacking interaction distance between the two adjacent layers of COF_TTA-DHTA_ (**h**).

Firstly, Fourier transform infrared spectra (FTIR) indicating the N–H bond of TTA (3210 cm^−1^, 3320 cm^−1^, and 3460 cm^−1^) and the CHO bond of DHTA (1670 cm^−1^) disappeared while the C=N bond (1623 cm^−1^) emerged (Figure 1a), demonstrating the successful synthesis of COF_TTA-DHTA_ [41,42]. After epoxy functionalization, the hydroxyl band of COF_TTA-DHTA_ at 1317 cm^−1^ diminished while a new peak emerged at 1290 cm^−1^, which was caused by the stretching vibration of the epoxy functional group C–O–C [35]. The results preliminarily indicated the –OH of COF_TTA-DHTA_ was successfully replaced by the epoxy functional group. The N_2_ adsorption and desorption isothermal test of COF_TTA-DHTA_ indicated that the Barrett–Emmett–Teller (BET) surface area of COF_TTA-DHTA_ was 89.4 m^2^/g and the pore size was 3.3 nm (Figure 1b). Compared with COF_TTA-DHTA_, the BET surface area of EP-COF_TTA-DHTA_ decreases significantly to 22.1 m^2^/g (Figure 1c). This decrease might be ascribed to the fact that the pores of COF_TTA-DHTA_ were blocked owing to the substitution of hydroxyl groups by epoxy functional groups, which further suggests the production of EP-COF_TTA-DHTA_.

The atomic force microscope (AFM) image demonstrated that COF_TTA-DHTA_ comprised 2D nanosheets with a thickness of 1.63 nm (Figure 1d). The morphology of COF_TTA-DHTA_ remained basically unchanged after epoxy functionalization, and EP-COF_TTA-DHTA_ was also composed of 2D nanosheets and the thickness was 1.82 nm (Figure 1e). The results prove that epoxy functionalization had minimal effect on the morphology of COF_TTA-DHTA_. Ab_1_ with a height of about 3.7 nm was uniformly anchored to the surface of EP-COF_TTA-DHTA_. After the antibody was connected, the surface roughness of EP-COF_TTA-DHTA_ increased significantly, which intuitively indicated that Ab_1_ was modified successfully (Figure 1f). The powder X-ray diffraction (PXRD) pattern of COF_TTA-DHTA_ showed many peaks at 2.52°, 4.84°, 5.49° and 7.58°, which corresponded to (100), (200), (210) and (001), respectively (Figure 1g). The results confirmed that COF_TTA-DHTA_ with good crystallization was successfully prepared. Meanwhile, the COF_TTA-DHTA_ has a *P1* space group with a = b = 39.8 Å, c = 3.461 Å, α = β = 90°, γ = 120°, and the AA stacking was approximated with the experimental XRD. In addition, the 3D structure of COF_TTA-DHTA_ showed that it was a hexagonal topological structure, and the spacing between the π–π stacks of COF_TTA-DHTA_ was calculated to be 3.46 Å (Figure 1h).

### 3.2. Characterization of Ab_2_/AuNPs@COF_TTA-2,6-NA(OH)2_

**Figure 2:**
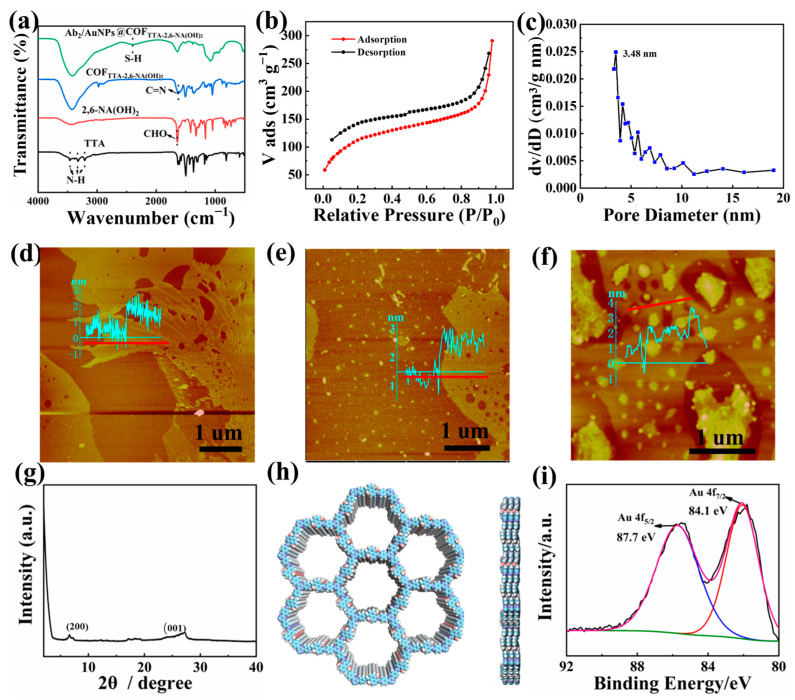
FTIR spectra of TTA (black), 2,6-NA(OH)_2_ (red), COF_TTA-2,6-NA(OH)2_ (blue) and Ab_2_/AuNPs@COF_TTA-2,6-NA(OH)2_ (green) (**a**). N_2_ adsorption and desorption isotherm of COF_TTA-2,6-NA(OH)2_ (**b**). Aperture distribution of COF_TTA-2,6-NA(OH)2_ (**c**). After being successively modified with AuNPs and Ab_2_, the roughness of the material surface of the AFM images of COF_TTA-2,6-NA(OH)2_ (**d**), AuNPs@COF_TTA-2,6-NA(OH)2_ (**e**) and Ab_2_/AuNPs@COF_TTA-2,6-NA(OH)2_ (**f**) accordingly (red line is the cross-sectional height). XRD pattern of COF_TTA-2,6-NA(OH)2_ (**g**). Eclipsed conformations of COF_TTA-2,6-NA(OH)2_ and the π–π stacking distance between the two adjacent layers of COF_TTA-2,6-NA(OH)2_ (**h**). The core-level spectra of Au (0) 4f of AuNPs@COF_TTA-2,6-NA(OH)2_ (**i**).

COF_TTA-2,6-NA(OH)2_ was used as a signal probe based on the abundant electroactive 2,6-NA(OH)_2_ parts (Figure S3). In the FTIR spectrum (Figure 2a), the N–H bond of TTA (3210 cm^−1^, 3319 cm^−1^ and 3458 cm^−1^) and the CHO bond of TTA-2,6-NA(OH)_2_ (1641 cm^−1^) disappeared while the C=N bond at 1623 cm^−1^ appeared, demonstrating the occurrence of the amino–aldehyde condensation reaction [43]. The characteristic Au-S peak at 2420 cm^−1^ appeared in the FTIR spectrum of Ab_2_/AuNPs@COF_TTA-2,6-NA(OH)2_, confirming that Ab_2_ was successfully grafted onto AuNPs@COF_TTA-2,6-NA(OH)2_. The N_2_ adsorption and desorption isothermal test showed that COF_TTA-2,6-NA(OH)2_ had a high BET surface area of 399.4 m^2^/g (Figure 2b) and that the pore size was 3.48 nm (Figure 2c).

Figure 2d shows that COF_TTA-2,6-NA(OH)2_ was a 2D nanosheet with a thickness of 1.97 nm and a smooth surface, which was conducive to the electrode and fully exposed its binding site for further loading of AuNPs and Ab_2_. After being modified with AuNPs, the nanoparticles with a height of about 1.5 nm were distributed on the surface of COF_TTA-2,6-NA(OH)2_ evenly, and the roughness of the material surface was increased slightly (Figure 2e). The results confirmed the successful preparation of AuNPs@COF_TTA-2,6-NA(OH)2_. In addition, particles were distributed on the 2D film, the roughness of the surface of AuNPs@COF_TTA-2,6-NA(OH)2_ further increased, and the size of the particles was significantly larger than the AuNPs (Figure 2f). The results confirmed that Ab_2_ was successfully fixed on the COF through powerful Au–S bonds.

The PXRD pattern showed diffraction peaks at 6.64° and 27.05°, which corresponded to (200) and (001) crystal planes, respectively, demonstrating that the COF_TTA-2,6-NA(OH)2_ material has a good crystal structure (Figure 2g). Furthermore, Figure 2h shows that the as-synthesized COF_TTA-2,6-NA(OH)2_ had a hexagonal topological structure, and the stacking layer spacing was 3.4 Å. Finally, the elements of Au@COF_TTA-2,6-NA(OH)2_ were analyzed by full X-ray photoelectron spectroscopy (XPS) (Figure S5). XPS analysis confirmed the successful reduction of Au (3+) to Au (0) nanoparticles uniformly distributed on the COF matrix. The material consisted of C, N, O and Au elements and the high-resolution spectrum of Au 4f showed two peaks at 87.7 eV and 84.1 eV, which corresponded to the peak of Au (0)-4f 5/2 and Au (0)-4f 7/2, respectively (Figure 2i). These results demonstrate that the AuNPs were modified successfully [44].

### 3.3. Electrochemical Behaviors

**Figure 3:**
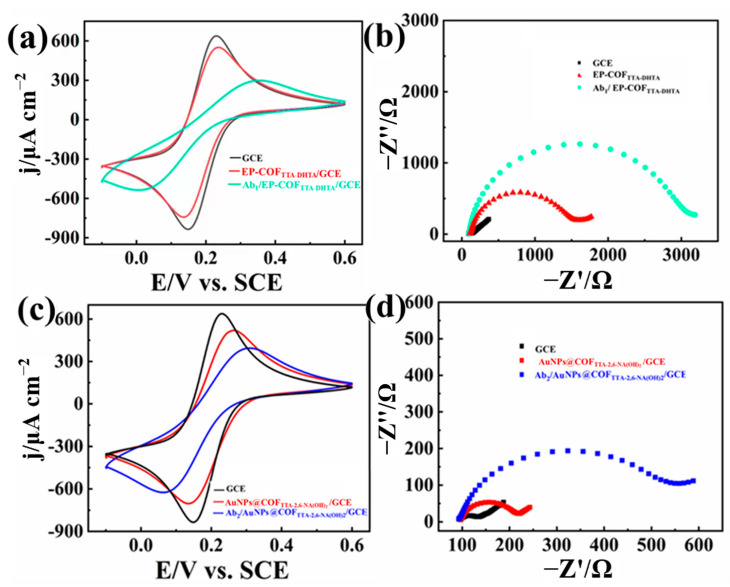
CV (**a**) and EIS (**b**) of GCE, EP-COF_TTA-DHTA_/GCE, and Ab_1_/EP-COF_TTA-DHTA_/GCE in 0.1 M KCl aqueous solution containing 5 mM [Fe (CN)6]^3−/4−^ at a scanning rate of 50 mV s^−1^. CV (**c**) and EIS (**d**) of GCE, AuNPs@COF_TTA-2,6-NA(OH)2_/GCE, and Ab_2_/AuNPs@COF_TTA-2,6-NA(OH)2_/GCE in 0.1 M KCl aqueous solution containing 5 mM [Fe (CN)6]^3−/4−^ at a scanning rate of 50 mV s^−1^. The consistency between the CV and EIS curves confirms the successful completion of each assembly step.

The establishment process of the electrochemical immunosensor was investigated with cyclic voltammetry (CV) and electrochemical impedance spectroscopy (EIS) in 0.1 M KCl solution containing 5 mM [Fe(CN)_6_]^3−/4−^. As shown in Figure 3a, GCE has a good pair of reversible redox peaks. After being modified with EP-COF_TTA-DHTA_, the peak current density of EP-COF_TTA-DHTA_/GCE decreased and the peak-to-peak potential difference increased slightly, which might be attributed to EP-COF_TTA-DHTA_ inhibiting the electron transport of [Fe(CN)_6_]^3/4−^. After Ab_1_ was further modified on EP-COF_TTA-DHTA_/GCE, the peak current density was sharply reduced due to biomacromolecules with a weak electrical conductivity that greatly hindered the electron transport of [Fe(CN)_6_]^3/4−^. The results proved the effective anchoring of Ab_1_ on EP-COF_TTA-DHTA_/GCE. The EIS also confirmed the above conclusion. After being modified by EP-COF_TTA-DHTA_, Ab_1_ was consecutively loaded onto the bare GCE, and the impedance value increased from 65 Ω to 1482 Ω and then to 2913 Ω (Figure 3b). CV and EIS were also suitable to prove the successful construction of an Ab_2_/AuNPs@COF_TTA-2,6-NA(OH)2_ immunosensor beacon. Figure 3c,d show that after the modification of AuNPs@COF_TTA-2,6-NA(OH)2_, the peak current density of AuNPs@COF_TTA-2,6-NA(OH)2_/GCE decreased and the EIS value increased slightly, which confirmed AuNPs@COF_TTA-2,6-NA(OH)2_ was feasible as the signal probe of the immunosensor. The peak current density decreased significantly after the Ab_2_ was further loaded, which indicated the Ab_2_ was modified on COF_TTA-2,6-NA(OH)2_/GCE successfully. The conclusion could be also confirmed by EIS on account of the impedance value rise from 59 Ω to 221 Ω and 548 Ω as the AuNPs@COF_TTA-2,6-NA(OH)2_ and Ab_2_ were subsequently modified on the electrode. As the materials with poor conductivity were modified step by step, the interlayer spacing increased and the mass transfer was blocked, which led to the gradually increased impedance value.

**Figure 4:**
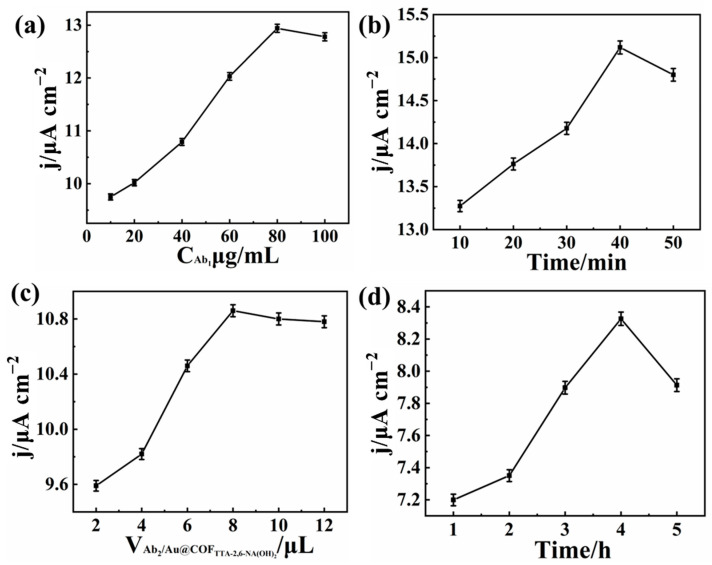
Effects of the concentration of Ab_1_ (**a**), incubating time of EP-COF_TTA-DHTA_/GCE in Ab_1_ (**b**), volume of Ab_2_/AuNPs@COF_TTA-2,6-NA(OH)2_ (**c**) and incubating time of Ab_2_/AuNPs@COF_TTA-2,6-NA(OH)2_/GCE (**d**) on the response of AuNPs@COF_TTA-2,6-NA(OH)2_/Ab_2_/CA 19-9/Ab_1_/EP-COF_TTA-DHTA_/GCE toward 100 U/mL CA 19-9.

To optimize the experimental conditions, the sandwich-type electrochemical immunosensor constructed under different experimental conditions was tested for 100 U/mL CA 19-9. Firstly, the concentration of Ab_1_ used to prepare Ab_1_/EP-COF_TTA-DHTA_/GCE was optimized. When the Ab_1_ concentration was increased from 10 μg/mL to 100 μg/mL, the current density increased significantly and then remained constant. With the concentration of Ab_1_ increased, the number of captured antigens also increased, which was conducive to the specific binding of the antigen and antibody, resulting in a higher current response. Therefore, the optimal concentration of Ab_1_ was 80 μg/mL (Figure 4a). Then, the incubation time of EP-COF_TTA-DHTA_/GCE in the Ab_1_ solution was optimized. As shown in Figure 4b, 40 min was selected as the optimum incubation time. Next, the volume of Ab_2_/AuNPs@COF_TTA-2,6-NA(OH)2_ was optimized. When the volume was 8 mL, the current response reached the maximum value (Figure 4c). As shown in Figure 4d, the optimum incubation time of Ab_2_/AuNPs@COF_TTA-2,6-NA(OH)2_ was 4 h.

### 3.4. Electrochemical Detection CA 19-9 Based on AuNPs@COF_TTA-2,6-NA(OH)2_/Ab_2_/CA 19-9/Ab_1_/EP-COF_TTA-DHTA/GCE_

**Figure 5:**
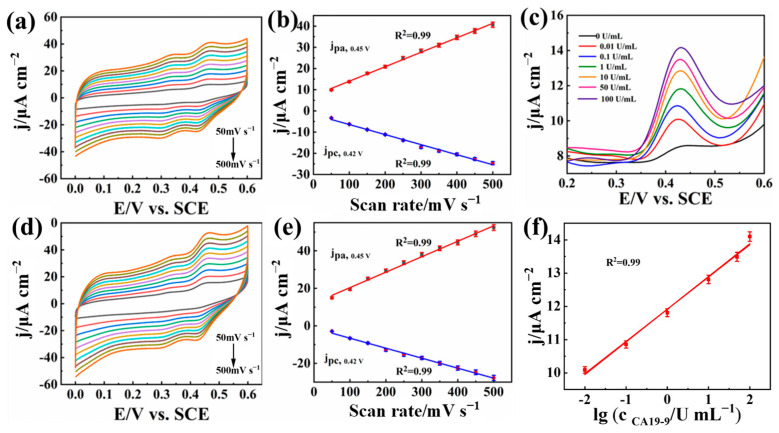
CVs of COF_TTA-2,6-NA(OH)2_ (**a**) and AuNPs@ COF_TTA-2,6-NA(OH)2_/GCE (**d**) in 0.1 M PBS (pH = 7) at different scanning rates. The plots of the redox peak currents of COF_TTA-2,6-NA(OH)2_/GCE (**b**) and AuNPs@COF_TTA-2,6-NA(OH)2_/GCE (**e**) versus the scanning rates (from black to orange, it represents an increasing scan rate from 50 to 500 mV s^−1^). DPV of AuNPs/COF_TTA-2,6-NA(OH)2/_Ab_2_/CA 19-9/Ab_1_/EP-COF_TTA-DHTA_/GCE in 0.2 M N_2_-saturated PBS (pH = 7) with different concentrations of CA 19-9 (**c**). The plots of current density versus lg*c*_CA 19-9_ (**f**).

The electrochemical parameters were calculated to explore the electrochemical capacity of the electrochemical immunosensor. In 0.2 M PBS (pH 7.0), the current density of COF_TTA-2,6-NA(OH)2_ (Figure 5a,b) and AuNPs@COF_TTA-2,6-NA(OH)2_ (Figure 5d,e) increased linearly with an increasing scan rate, and the oxidation peak of AuNPs@COF_TTA-2,6-NA(OH)2_ at 0.45 V was significantly higher than that of COF_TTA-2,6-NA(OH)2_ at the same scan rate, indicating that the modification of AuNPs could improve the conductivity of materials. According to Laviron’s theory, to investigate the properties of COF_TTA-2,6-NA(OH)2_ [45], the electron transfer coefficient αs was calculated to be 0.5, demonstrating the electrochemical process was a quasi-reversible surface adsorption control process. The electron transfer rate constant (ks) of COF_TTA-2,6-NA(OH)2_/GCE was 2.33 s^−1^, while the ks of AuNPs@COF_TTA-2,6-NA(OH)2_/GCE was 3.54 s^−1^ when the scan rate was 50 mV s^−1^. The results confirmed that AuNPs could attach Ab_2_ firmly and increase the electron transfer rate. The linear fitting diagram showed that the CA 19-9 immunosensor exhibited a wide linear concentration range of 0.0009–100 U/mL and a low detection limit of 0.0003 U/mL (Figure 5c,f).

Finally, the performance of the electrochemical immunosensor was compared with some previously reported biosensors (Table 1). For example, compared with electrochemical immunosensors constructed with AuPt nanocalliandras [46], the unique sandwich structure requires that Ab_1_ first captures the antigen and then binds to electroactive COFs loaded with Ab_2_, ensuring specific recognition of the analyte, reducing the interference from other substances and eliminating the need to incorporate additional electroactive substances. The result indicated it was better than most other reported CA 19-9 electrochemical immunosensors.

**Figure 6:**
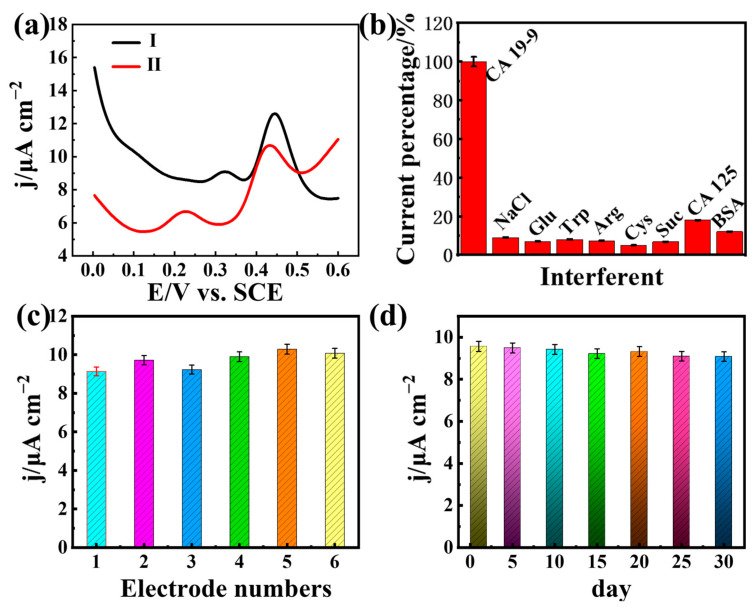
DPV of AuNPs@COF_TTA-2,6-NA(OH)2_ (I) and AuNPs@COF_TTA-2,6-NA(OH)2_/Ab_2_/CA 19-9/Ab_1_/EP-COF_TTA-DHTA_/GCE (II) in 0.2 M N_2_-saturated PBS (pH = 7) with 10 U/mL CA 19-9 (**a**). Selectivity of AuNPs@COF_TTA-2,6-NA(OH)2_/Ab_2_/CA 19-9/Ab_1_/EP-COF_TTA-DHTA_/GCE toward substances that might coexist with CA 19-9 in the concentration of 50 ng/mL in 0.2 M N_2_-saturated PBS (pH = 7) (**b**). Current responses of six different AuNPs@COF_TTA-2,6-NA(OH)2_/Ab_2_/CA 19-9/Ab_1_/EP-COF_TTA-DHTA_/GCE to 10 U/mL CA 19-9 (**c**) (different colors represent different parallel electrodes). Current responses of AuNPs@COF_TTA-2,6-NA(OH)2_/Ab_2_/CA 19-9/Ab_1_/EP-COF_TTA-DHTA_/GCE to 10 U/mL CA 19-9 after different days (**d**) (different colors represent different days).

Figure 6a shows that COF_TTA-2,6-NA(OH)2_ had a significant oxidation peak in N_2_-saturated PBS at 0.45 V, while the oxidation peak of AuNPs@COF_TTA-2,6-NA(OH)2_/Ab_2_/CA 19-9/Ab_1_/EP-COF_TTA-DHTA_/GCE (for detection of 10 U/mL CA 19-9) appeared at 0.42 V. The decrease in the oxidation peak potential might be owing to the AuNPs’ enhanced electron transfer. To investigate the anti-interference ability of the electrochemical immunosensor, 10 U/mL CA 19-9 was detected in N_2_-saturated PBS with 50 ng/mL interferents (including NaCl, glucose, tryptophan (Trp) arginine (Arg), cysteine (Cys), sucrose (Suc), and CA125) that may coexist with CA 19-9 in blood (Figure 6b). The results demonstrated that these substances have less interference with the CA 19-9 sensor. In addition, the repeatability and stability of the electrochemical immunosensor were also tested. Six different AuNPs@COF_TTA-2,6-NA(OH)2_/Ab_2_/CA 19-9/Ab_1_/EP-COF_TTA-DHTA_/GCE were prepared for the detection of 10 U/mL CA 19-9 with a relative standard deviation of about 1.43%. The results demonstrated the constructed electrochemical immunosensor exhibited excellent repeatability (Figure 6c). Subsequently, the same AuNPs@COF_TTA-2,6-NA(OH)2_/Ab_2_/CA 19-9/Ab_1_/EP-COF_TTA-DHTA_/GCE was tested three times every five days, and the current density only dropped by 9.80% after 35 days, which proved that the electrochemical immunosensor also possessed good stability (Figure 6d). Moreover, the recovery rate of the diluted human serum samples was 96.5~103.8% (Table S1), and the results obtained by the proposed assay were highly consistent with traditional methods used to detect clinical samples (Table S2), proving that the electrochemical immunosensor has a promising application prospect in clinical practice diagnostics.

## 4. Conclusions

In this work, a sandwich-type electrochemical immunosensor based on two kinds of 2D COFs to ultra-sensitively detect CA 19-9 was reported. To enhance the stability and sensitivity of the sensor, 2D COFs, as excellent carriers of immobilizing biomolecules, provide a friendly platform with a large number of effective binding sites. Epoxy-functionalized EP-COF_TTA-DHTA_ and Ab_1_ were connected stably through covalent bonds and the clever design of electroactive COF_TTA-2,6-NA(OH)2_ for the effective binding of Ab_2_ through Au–S bonds. After the electroactive COF_TTA-2,6-NA(OH)2_ was loaded with AuNPs, the electrochemical signal was further amplified and the sensitivity of the immunosensor was improved accordingly. The prepared AuNPs@COF_TTA-2,6-NA(OH)2_/Ab_2_/CA 19-9/Ab_1_/EP-COF_TTA-DHTA_-based electrochemical immunosensor had a low LOD (0.0003 U/mL), wide linear range (0.0009–100 U/mL), good reproducibility, excellent stability and good selectivity. This method provides an important reference for the development of the clinical detection of CA 19-9 sensors.

## Figures and Tables

**Table 1:** Comparison between the proposed assay and other methods for detection of C19-9A.

Materials	Analytical Technique	LOD (U/mL))	Linear Range (U/mL)	Matrix	Refs
AuPt nanocalliandras	DPV	0.03	0.05–50	PBS serum	[46]
Antibody-AuNP-G-quadruplex/hemin	CLIA	0.016	0.025–1.00	PBS serum	[47]
Cuboid silver-modified paper working electrode	SWV	0.00004	0.1–100	PBS serum	[48]
MWCNT-Pt-Luminol	ECL	0.046 × 10^−3^	0.01–10	PBS serum	[49]
Au/GO-MA	DPV	0.032	0.01–100	PBS serum	[50]
CB	DPV	0.07	0.01–40	PBS serum	[51]
Chitosan	CL	1	2.0–25	PBS serum	[52]
Multi-functionalized graphene oxide	ECL	0.0005	0.001–5	serum urine	[53]
AuNPs@COF_TTA-2,6-NA(OH)2_/Ab_2_/CA 19-9/Ab_1_/EP-COF_TTA-DHTA_	DPV	0.0003	0.0009–100	PBS, serum	This work

## Data Availability

The original contributions presented in this study are included in the article; further inquiries can be directed to the corresponding author.

## Supplementary Materials

The following supporting information can be downloaded at https://www.mdpi.com/article/10.3390/bios15080492/s1. Figure S1. Schematic illustration of COF_TTA-DHTA_ synthesis. Figure S2. Schematic illustration of EP-COF_TTA-DHTA_ synthesis. Figure S3. Schematic illustration of COF_TTA-2,6-NA(OH)2_ synthesis. Figure S4. Chemical structural unit of Ab_1_/EP-COF_TTA-DHTA_. Figure S5. XPS survey spectra of AuNPs@COF_TTA-2,6-NA(OH)2_. Table S1. Determination of CA 19-9 in human serum samples. Table S2. Performance comparison of the proposed assay with conventional methods (Immunoradiometricassay) for detecting clinical samples.

## References

[1] Wang M., Hu M., Hu B., Guo C., Song Y., Jia Q., He L., Zhang Z., Fang S. (2019). Bimetallic cerium and ferric oxides nanoparticles embedded within mesoporous carbon matrix: Electrochemical immunosensor for sensitive detection of carbohydrate antigen 19-9. Biosens. Bioelectron..

[2] An Y., Song L., Chen X., Ni C., Mao K., Zhu L., Gu Y., Miao Y., Song B., Ma H. (2022). 3D Biomimetic hydrangea-like biOCl and PtNi nanocube-based electrochemical immunosensor for quantitative detection of CA19-9. J. Electrochem. Soc..

[3] Chang J., Lv W., Wu J., Li H., Li F. (2021). Simultaneous photoelectrochemical detection of dual microRNAs by capturing CdS quantum dots and methylene blue based on target-initiated strand displaced amplification. Chin. Chem. Lett..

[4] Liu X., Li X., Gao X., Ge L., Sun X., Li F. (2019). A universal paper-based electrochemical sensor for zero-background assay of diverse biomarkers. ACS Appl. Mater. Interfaces.

[5] Zhao Y., Xiang J., Cheng H., Liu X., Li F. (2021). Flexible photoelectrochemical biosensor for ultrasensitive microRNA detection based on concatenated multiplex signal amplification. Biosens. Bioelectron..

[6] Luo K., Zhao C., Luo Y., Pan C., Li J. (2022). Electrochemical sensor for the simultaneous detection of CA72-4 and CA19-9 tumor markers using dual recognition via glycosyl imprinting and lectin-specific binding for accurate diagnosis of gastric cancer. Biosens. Bioelectron..

[7] Li B., Li Y., Li C., Yang J., Liu D., Wang H., Xu R., Zhang Y., Wei Q. (2023). An ultrasensitive split-type electrochemical immunosensor based on controlled-release strategy for detection of CA19-9. Biosens. Bioelectron..

[8] Li H., Shao M., Fang J., Li Y., Sun X., Ren X., Wei Q., Ju H., Ma H. (2024). Metal-Organic framework incorporated luminescent PTCA combined with novel co-reactant accelerator for ultra-sensitive electrochemiluminescence detection of CA 19-9. Chem. Eng. J..

[9] Mo G., He X., Qin D., Meng S., Wu Y., Deng B. (2021). Spatially-resolved dual-potential sandwich electrochemiluminescence immunosensor for the simultaneous determination of carbohydrate antigen 19-9 and carbohydrate antigen 24-2. Biosens. Bioelectron..

[10] Lee H.S., Jang C.Y., Kim S.A., Park S.B., Jung D.E., Kim B.O., Kim H.Y., Chung M.J., Park J.Y., Bang S. (2018). Combined use of CEMIP and CA 19-9 enhances diagnostic accuracy for pancreatic cancer. Sci. Rep..

[11] Satake K., Takeuchi T. (1994). Comparison of CA19-9 with other tumor markers in the diagnosis of cancer of the pancreas. Pancreas.

[12] Ilie-Mihai R.-M., Stefan-van Staden R.-I., van Staden J.K.F. (2022). Progress in electroanalysis of p53, CEA, and CA19-9. J. Electrochem. Soc..

[13] Tan Y., Wei X., Zhang Y., Wang P., Qiu B., Guo L., Lin Z., Yang H.-H. (2015). Exonuclease-catalyzed target recycling amplification and immobilization-free electrochemical aptasensor. Anal. Chem..

[14] Chen Y., Xianyu Y., Jiang X. (2017). Surface modification of gold nanoparticles with small molecules for biochemical analysis. Acc. Chem. Res..

[15] Sha F., Xie H., Son F.A., Kim K.S., Gong W., Su S., Ma K., Wang X., Wang X., Farha O.K. (2023). Rationally tailored mesoporous hosts for optimal protein encapsulation. J. Am. Chem. Soc..

[16] Zheng Y., Li J., Zhou B., Ian H., Shao H. (2021). Advanced sensitivity amplification strategies for voltammetric immunosensors of tumor marker: State of the art. Biosens. Bioelectron..

[17] Li F., Zhang H., Wang Z., Newbigging A.M., Reid M.S., Li X.-F., Le X.C. (2015). Aptamers facilitating amplified detection of biomolecules. Anal. Chem..

[18] Gu C., Kong X., Liu X., Gai P., Li F. (2019). Enzymatic biofuel-cell-based self-powered biosensor integrated with DNA amplification strategy for ultrasensitive detection of single-nucleotide polymorphism. Anal. Chem..

[19] Ning S., Zhou M., Liu C., Waterhouse G.I., Dong J., Ai S. (2019). Ultrasensitive electrochemical immunosensor for avian leukosis virus detection based on a β-cyclodextrin-nanogold-ferrocene host-guest label for signal amplification. Anal. Chim. Acta.

[20] Chen H., Li Y., Song Y., Liu F., Deng D., Zhu X., He H., Yan X., Luo L. (2023). A sandwich-type electrochemical immunosensor based on spherical nucleic acids-templated Ag nanoclusters for ultrasensitive detection of tumor biomarker. Biosens. Bioelectron..

[21] Liang H., Luo Y., Xiao Y., Xiong J., Chen R., Song Y., Wang L. (2023). Immunosensing of neuron-specific enolase based on dual signal amplification strategy via electrocatalytic oxygen reduction by iron-porphyrin covalent organic framework. Chem. Eng. J..

[22] Naberezhnyi D., Park S., Li W., Westphal M., Feng X., Dong R., Dementyev P. (2021). Mass transfer in boronate ester 2D COF single crystals. Small.

[23] Wang W., Zhao W., Xu H., Liu S., Huang W., Zhao Q. (2021). Fabrication of ultra-thin 2D covalent organic framework nanosheets and their application in functional electronic devices. Coord. Chem. Rev..

[24] Sun X., Wang N., Xie Y., Chu H., Wang Y., Wang Y. (2021). In-situ anchoring bimetallic nanoparticles on covalent organic framework as an ultrasensitive electrochemical sensor for levodopa detection. Talanta.

[25] Skorjanc T., Shetty D., Valant M. (2021). Covalent organic polymers and frameworks for fluorescence-based sensors. ACS Sens..

[26] Zhang K., Kirlikovali K.O., Varma R.S., Jin Z., Jang H.W., Farha O.K., Shokouhimehr M. (2020). Covalent organic frameworks: Emerging organic solid materials for energy and electrochemical applications. ACS Appl. Mater. Interfaces.

[27] Babu H.V., Bai M.M., Rajeswara Rao M. (2019). Functional π-conjugated two-dimensional covalent organic frameworks. ACS Appl. Mater. Interfaces.

[28] Pan F., Tong C., Wang N., Wang Y., Pan D., Zhu R. (2022). Adsorbent-assisted in situ electrocatalysis: Highly sensitive and stable electrochemical sensor based on AuNF/COF-SH/CNT nanocomposites for the determination of trace Cu (II). ACS Sustain. Chem. Eng..

[29] Sahoo R., Mondal S., Pal S.C., Mukherjee D., Das M.C. (2021). Covalent-organic frameworks (COFs) as proton conductors. Adv. Energy Mater..

[30] Liu T.-Z., Hu R., Liu Y., Zhang K.-L., Bai R.-Y., Yang Y.-H. (2020). Amperometric immunosensor based on covalent organic frameworks and Pt/Ru/C nanoparticles for the quantification of C-reactive protein. Microchim. Acta.

[31] Feng S., Yan M., Xue Y., Huang J., Yang X. (2021). Electrochemical immunosensor for cardiac troponin I detection based on covalent organic framework and enzyme-catalyzed signal amplification. Anal. Chem..

[32] Bu T., Bai F., Sun X., Tian Y., Zhang M., Zhao S., He K., Wang X., Jia P., Wang L. (2021). An innovative prussian blue nanocubes decomposition-assisted signal amplification strategy suitable for competitive lateral flow immunoassay to sensitively detect aflatoxin B1. Food Chem..

[33] Guo Q., Ding L., Li Y., Xiong S., Fang H., Li X., Nie L., Xiong Y., Huang X. (2022). Covalent organic framework-gold nanoparticle heterostructures amplified dynamic light scattering immunosensor for ultrasensitive detection of NT-proBNP in whole blood. Sens. Actuators B Chem..

[34] Zhang X., Gao Y., Li J., Yan J., Liu P., Fan X., Song W. (2023). A novel TAPP-DHTA COF cathodic photoelectrochemical immunosensor based on CRISPR/Cas12a-induced nanozyme catalytic generation of heterojunction. Electrochim. Acta.

[35] Xing C., Mei P., Mu Z., Li B., Feng X., Zhang Y., Wang B. (2022). Enhancing enzyme activity by the modulation of covalent interactions in the confined channels of covalent organic frameworks. Angew. Chem..

[36] Yang Y., Li G., Wang P., Fan L., Shi Y. (2022). Highly sensitive multiplex detection of foodborne pathogens using a SERS immunosensor combined with novel covalent organic frameworks based biologic interference-free Raman tags. Talanta.

[37] Wu L., Chen R., Jin W., Peng C., Wang L., Miao L., Song Y. (2025). An electrochemical immunosensor for carbohydrate antigen CA125 based on electroactive COFs as a signal probe. Talanta.

[38] Jin W., Chen R., Wu L., Peng C., Song Y., Miao L., Wang L. (2025). An “on-off” electrochemical immunosensor for the detection of the glycan antigen CA125 by amplification signals using electropositive COFs. Talanta.

[39] Liang H., Xiao Y., Chen R., Li Y., Zhou S., Liu J., Song Y., Wang L. (2023). Immunosensing of neuron-specific enolase based on signal amplification strategies via catalysis of ascorbic acid by heteropolysate COF. Biosens. Bioelectron..

[40] Wang J., Guo Z., Xiong J., Wu D., Li S., Tao Y., Qin Y., Kong Y. (2019). Facile synthesis of chitosan-grafted beta-cyclodextrin for stimuli-responsive drug delivery. Int. J. Biol. Macromol..

[41] Zhang T., Chen Y., Huang W., Wang Y., Hu X. (2018). A novel AuNPs-doped COFs composite as electrochemical probe for chlorogenic acid detection with enhanced sensitivity and stability. Sens. Actuators B Chem..

[42] Xu M., Wang L., Xie Y., Song Y., Wang L. (2019). Ratiometric electrochemical sensing and biosensing based on multiple redox-active state COF_DHTA-TTA_. Sens. Actuators B Chem..

[43] Yang T.-L., Chen J.-Y., Kuo S.-W., Lo C.-T., El-Mahdy A.F.M. (2022). Hydroxyl-functionalized covalent organic frameworks as high-performance supercapacitors. Polymers.

[44] Deng Y., Zhang Z., Du P., Ning X., Wang Y., Zhang D., Liu J., Zhang S., Lu X. (2020). Embedding ultrasmall Au clusters into the pores of a covalent organic framework for enhanced photostability and photocatalytic performance. Angew. Chem..

[45] Laviron E. (1979). General expression of the linear potential sweep voltammogram in the case of diffusionless electrochemical systems. J. Electroanal. Chem. Interfacial Electrochem..

[46] Weng X., Liu Y., Xue Y., Wang A.-J., Wu L., Feng J.-J. (2017). L-Proline bio-inspired synthesis of AuPt nanocalliandras as sensing platform for label-free electrochemical immunoassay of carbohydrate antigen 19-9. Sens. Actuators B Chem..

[47] Shi M., Zhao S., Huang Y., Zhao L., Liu Y.-M. (2014). Signal amplification in capillary electrophoresis based chemiluminescent immunoassays by using an antibody–gold nanoparticle–DNAzyme assembly. Talanta.

[48] Li W., Li L., Ge S., Song X., Ge L., Yan M., Yu J. (2014). Multiplex electrochemical origami immunodevice based on cuboid silver-paper electrode and metal ions tagged nanoporous silver–chitosan. Biosens. Bioelectron..

[49] Zhang X., Ke H., Wang Z., Guo W., Zhang A., Huang C., Jia N. (2017). An ultrasensitive multi-walled carbon nanotube–platinum–luminol nanocomposite-based electrochemiluminescence immunosensor. Analyst.

[50] Zhang N., Zhang D., Chu C., Ma Z. (2020). Label-assisted chemical adsorption triggered conversion of electroactivity of sensing interface to achieve the Ag/AgCl process for ultrasensitive detection of CA 19-9. Anal. Chim. Acta.

[51] Ibáñez-Redín G., Materon E.M., Furuta R.H.M., Wilson D., do Nascimento G.F., Melendez M.E., Carvalho A.L., Reis R.M., Oliveira O.N., Gonçalves D. (2020). Screen-printed electrodes modified with carbon black and polyelectrolyte films for determination of cancer marker carbohydrate antigen 19-9. Microchim. Acta.

[52] Lin J., Yan F., Hu X., Ju H. (2004). Chemiluminescent immunosensor for CA19-9 based on antigen immobilization on a cross-linked chitosan membrane. J. Immunol. Methods.

[53] Sha Y., Guo Z., Chen B., Wang S., Ge G., Qiu B., Jiang X. (2015). A one-step electrochemiluminescence immunosensor preparation for ultrasensitive detection of carbohydrate antigen 19-9 based on multi-functionalized graphene oxide. Biosens. Bioelectron..

