# Supplementary material for: A Sensitive Sandwich-Type Electrochemical Immunosensor for Carbohydrate Antigen 19-9 Based on Covalent Organic Frameworks
Source: Biosensors (Basel). 2025 Aug 1;15(8):492. doi: 10.3390/bios15080492 (PMC12384156; doi:10.3390/bios15080492)
Supplement: Supplementary file 1 [file biosensors-15-00492-s001.zip › biosensors-3751821-supplementary.pdf]

Supplementary Information

A sensitive sandwich-type electrochemical immunosensor for carbohydrate antigen 19-9 based on Covalent Organic frameworks

Ting Wu[1,2], Rongfang Chen[1], Yaqin Duan[1], Longfei Miao[1], Yongmei Zhu[1,*] and Li Wang[1,*]

[1]*College of Chemistry and Materials, Jiangxi Normal University, Nanchang 330022, China.*

[2]*School of New Energy Science and Engineering, Xinyu University, Xinyu, 338004, China*

Chemical reagents: 1,3,5-tris(4-aminophenyl) triazine (TTA) 2,6-dihydroxynaphthalene-1,5-dicarbaldehyde (2,6-NA$(OH)_2$) and 2,5-dihydroxyl-terephthalaldehyde (DHTA) were purchased from Academy of Sciences-Yanshen Technology Co., Ltd (Jilin, China). $HAuCl_4$ $4H_2O$, $NaBH_4$, epibromohydrin, trisodium citrate dihydrate, glucose, NaCl, tryptophan (Trp) arginine (Arg), cysteine (Cys) and sucrose (Suc) were obtained from Aladdin (Shanghai, China). Carbohydrate antigen 19-9 (CA 19-9), Anti-CA 19-9McAb (coating) (Ab_1), Anti- CA 19-9 McAb (Ab_2), *o*-dichlorobenzene (*o*-DCB), *n*-butyl alcohol (*n*-BuOH), acetic acid (HAc), N, N-dimethylformamide (DMF), K_2CO_3, Epibromohydrin and tetrahydrofuran (THF) were acquired from Innochem Co., Ltd (Beijing, China). Bovine serum albumin (BSA) was purchased from Beijing Solarbio science & technology Co., Ltd (Beijing, China). 0.2 M N_2-saturated phosphate buffer solution (PBS, pH 7) was prepared by 0.2 M NaH_2PO_4 and 0.2 M Na_2HPO_4. All solutions were prepared with ultra-pure water purified by a Millipore-Q System ($\rho \geq 18.2$ MΩ cm). Glassy carbon electrode (GCE) was obtained from Shanghai Chenhua Co., Ltd. (Shanghai, China). Bare GCE was firstly polished with 0.5 μm and 0.03 μm of Al_2O_3 polishing powder, respectively, and then was ultrasonic cleaned with ethanol and water. Finally the polished GCE were blow-dried with high purity nitrogen to obtain a clean, dry and smooth surface.

Instruments. All electrochemical experiments were conducted on the CHI-760E electrochemical workstation (Shanghai, China). Three-electrode system consisted of a saturated calomel electrode (SCE) as the reference electrode, a platinum wire electrode as the counter electrode and the modified electrode using as the working electrode. Cyclic voltammetry (CV) and electrochemical impedance spectroscopy (EIS) with frequency range varied from 0.01 Hz to 105 Hz were performed in 5.0 mM $Fe(CN)_6^{3-/4-}$ solution containing 0.1 M KCl. Differential pulse voltammetry (DPV) whose amplitude and pulse width were controlled respectively at 50 mV and 0.2 s were carried out in 0.2 M N_2-saturated PBS. Transmission electron microscopy (TEM) images were obtained via JEOL JEM-2100 microscopes at an acceleration voltage of 200 KV. Atomic force microscopy (AFM) image was collected by Bruker Nano-scope V (MultiMode 8) with ScanAsyst mode under atmosphere. N_2 adsorption-desorption isothermal test was carried out by Autosorb-iQ (Quantachrome) under a liquid nitrogen environment (77 K). D/Max 2500 V/PCX-ray powder diffract meter (PXRD) was conducted using Cu Kα radiation with range from 2° to 40° and scanning step maintained 3°/min. Fourier transform infrared spectroscopy (FTIR) was recorded on a Nicolet 6700 FT-IR spectrophotometer.

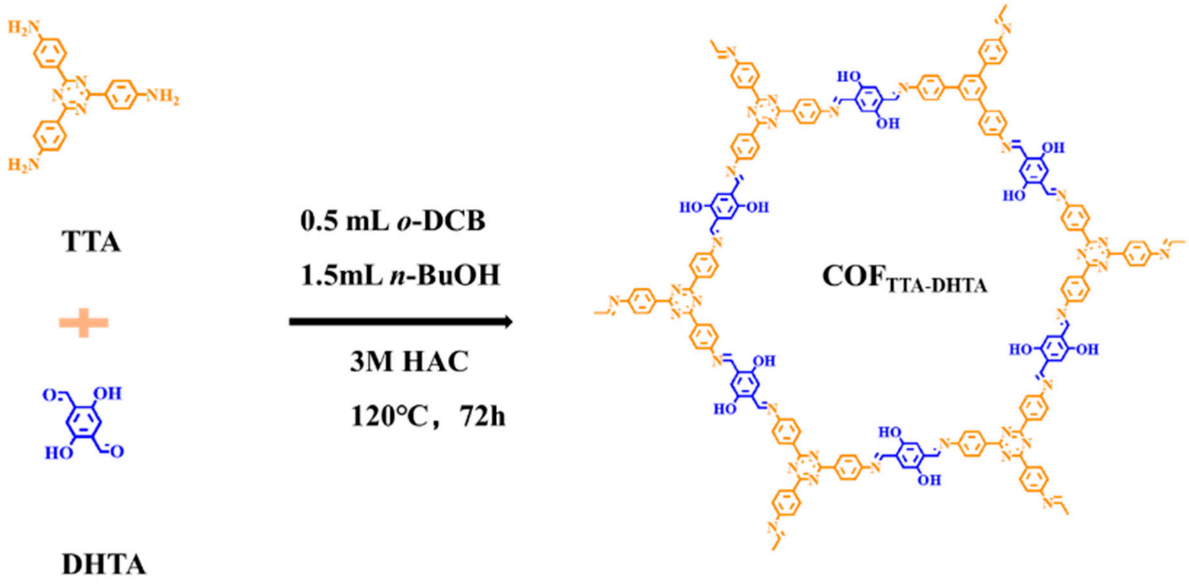

Figure S1. Schematic illustration of $COF_{TTA-DHTA}$ synthesis.

$COF_{TTA-DHTA}$ was prepared via the following procedure as shown in Fig. S1. 10.6 mg 1,3,5-triazine-2,4,6-triamine (TTA) and 7.47 mg 2,5-dihydroxy-1,4-benzenedicarboxaldehyde (DHTA) were added to a mixture of 1.5 mL *n*-butanol and 0.5 mL *o*-dichlorobenzene, ultrasonically dispersed, and transferred to 25 mL Teflon reaction tank. Then, 400 μL of 3 M HAC was added quickly as a catalyst and the reaction was maintained at 120 °C in an oven for 3 d. The product was cooled to room temperature and then centrifugally cleaned with tetrahydrofuran, acetone and dichloromethane successively. Finally, the product was dried in freeze-dryer.

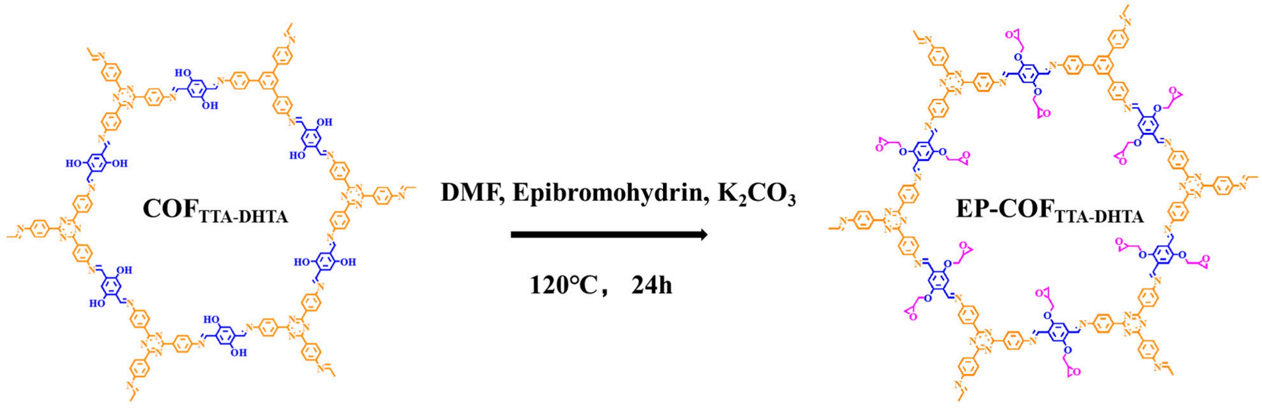

Figure S2. Schematic illustration of EP-COF$_{TTA-DHTA}$ synthesis.

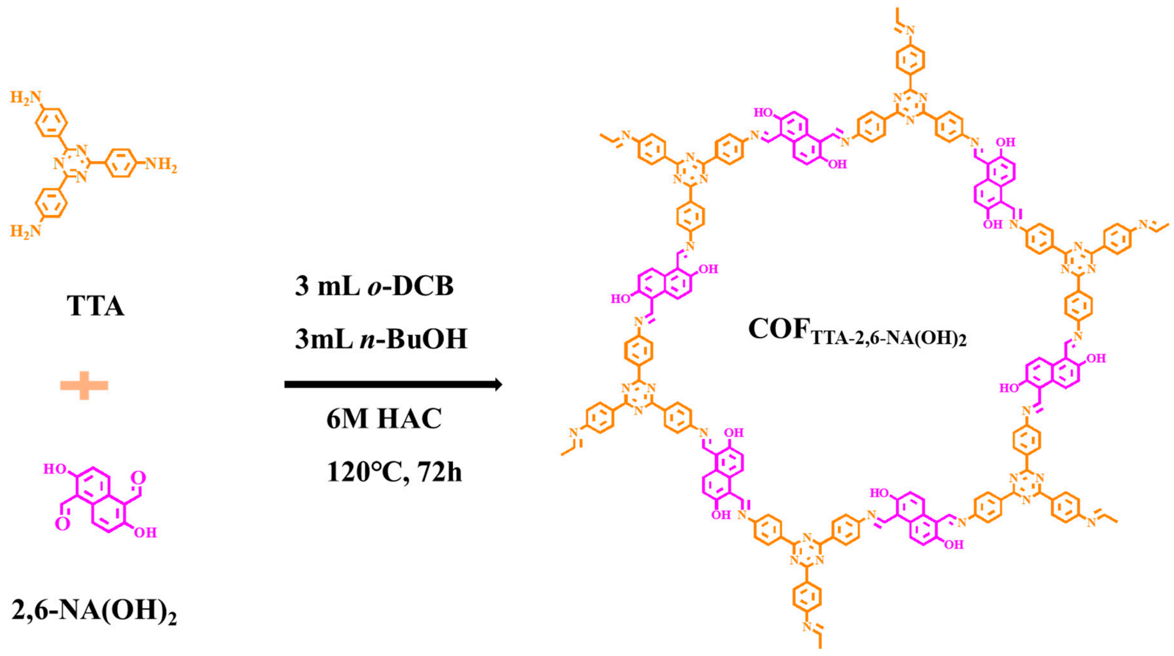

Figure S3. Schematic illustration of $COF_{TTA\text{-}2,6\text{-}NA(OH)2}$ synthesis.

$COF_{TTA\text{-}2,6\text{-}NA(OH)2}$ was prepared via the following procedure (Fig. S3). TTA (70.0 mg, 0. 2 mmol) and 2,6-NA$(OH)_2$ (64.7 mg, 0.3 mmol) were added to a mixed solution containing 3.5 mL *n*-butanol and 3.5 mL *o*-dichlorobenzene. After ultrasonic dispersion, 0.7 mL 6 M HAC was added as a catalyst and then transferred to 25 mL Schlenk storage tube. After three freezing/pumping/thawing, the tube was heated to 120 ºC for 3 d. After cooling to room temperature, the product was centrifugally cleaned with THF and DMF until the solution was clear. Finally, the product was dried for 12 h.

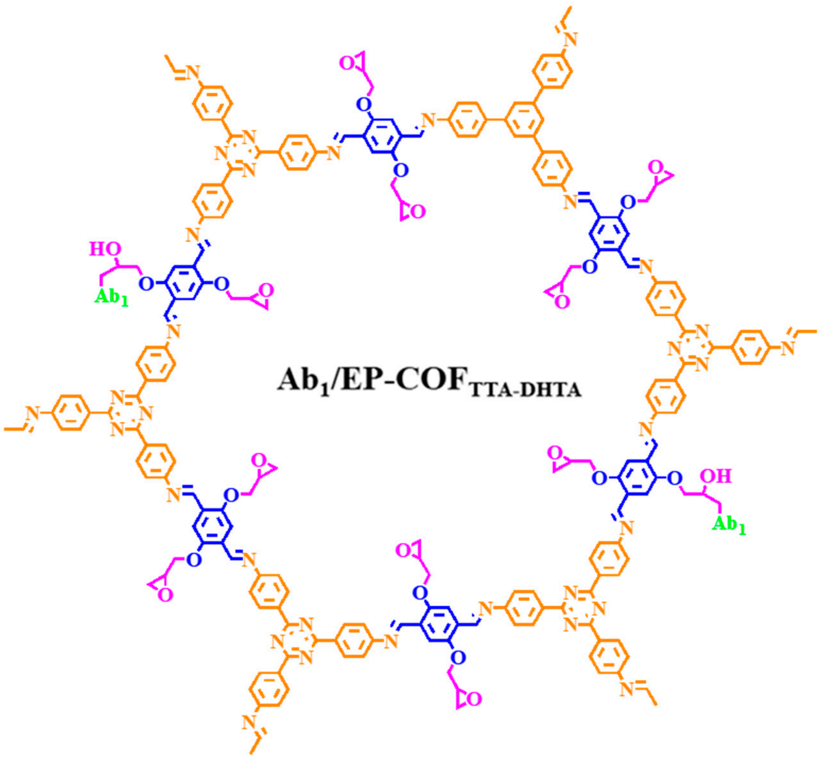

Figure S4. Chemical structural unit of Ab_1/EP-$COF_{TTA-DHTA}$

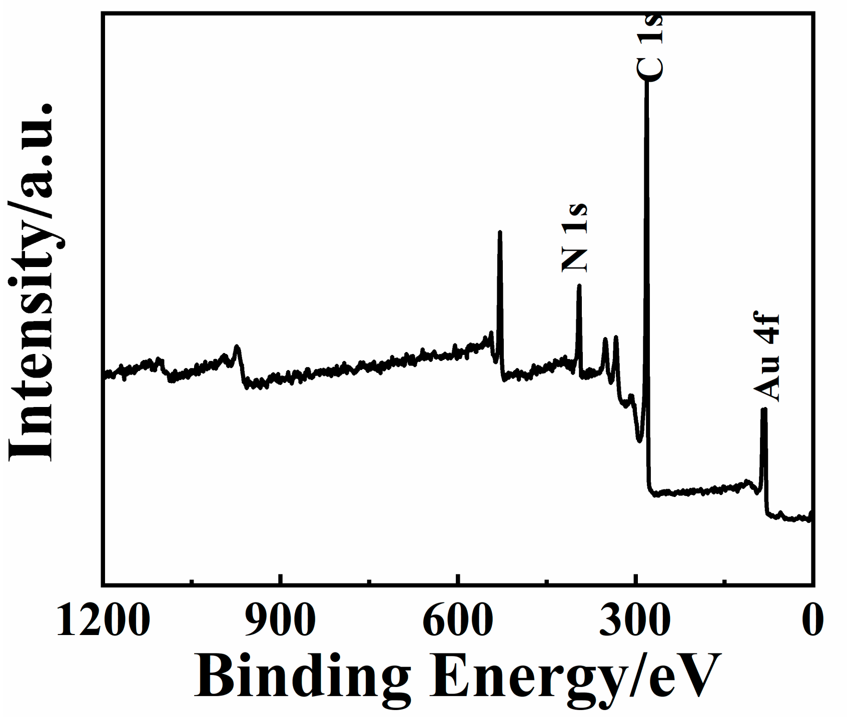

Figure S5. XPS survey spectra of AuNPs@$COF_{TTA-2,6-NA(OH)2}$.

Table S1. Determination of CA 19-9 in human serum samples

Human serum sample (U/mL)	Added (U/mL)	Measured value (U/mL)	Recovery (%)	RSD (%, n=3)
10	10	19.3	96.5	3.50
	20	30.7	102.3	2.33
	30	41.5	103.8	3.75

Table S2. Performance comparison of the proposed assay with conventional methods (Immunoradiometricassay) for detecting clinical samples

clinical samples	proposed method (U/mL)	Immunoradiometricassay (U/mL)
1	0.13	0.12
2	3.98	4.20
3	17.05	16.29
4	61.32	63.07
5	90.81	88.75

References

1. Weng, X.; Liu, Y.; Xue, Y.; Wang, A.-J.; Wu, L.; Feng, J.-J. L-Proline bio-inspired synthesis of AuPt nanocalliandras as sensing platform for label-free electrochemical immunoassay of carbohydrate antigen 19-9. *Sens, and Actuators B Chem.* **2017**, *250*, 61-68.
2. Shi, M.; Zhao, S.; Huang, Y.; Zhao, L.; Liu, Y.-M. Signal amplification in capillary electrophoresis based chemiluminescent immunoassays by using an antibody–gold nanoparticle–DNAzyme assembly. *Talanta* **2014**, *124*, 14-20.
3. Li, W.; Li, L.; Ge, S.; Song, X.; Ge, L.; Yan, M.; Yu, J. Multiplex electrochemical origami immunodevice based on cuboid silver-paper electrode and metal ions tagged nanoporous silver–chitosan. *Biosens. Bioelectron.* **2014**, *56*, 167-173.
4. Zhang, X.; Ke, H.; Wang, Z.; Guo, W.; Zhang, A.; Huang, C.; Jia, N. An ultrasensitive multi-walled carbon nanotube–platinum–luminol nanocomposite-based electrochemiluminescence immunosensor. *Analyst* **2017**, *142*, 2253-2260.
5. Zhang, N.; Zhang, D.; Chu, C.; Ma, Z. Label-assisted chemical adsorption triggered conversion of electroactivity of sensing interface to achieve the Ag/AgCl process for ultrasensitive detection of CA 19-9. *Anal. Chim. Acta* **2020**, *1093*, 43-51.
6. Ibáñez-Redín, G.; Materon, E.M.; Furuta, R.H.; Wilson, D.; do Nascimento, G.F.; Melendez, M.E.; Carvalho, A.L.; Reis, R.M.; Oliveira, O.N.; Gonçalves, D. Screen-printed electrodes modified with carbon black and polyelectrolyte films for determination of cancer marker carbohydrate antigen 19-9. *Microchim. Acta* **2020**, *187*, 1-11.
7. Lin, J.; Yan, F.; Hu, X.; Ju, H. Chemiluminescent immunosensor for CA19-9 based on antigen immobilization on a cross-linked chitosan membrane. *J. of Immunol. Methods* **2004**, *291*, 165-174.
8. Sha, Y.; Guo, Z.; Chen, B.; Wang, S.; Ge, G.; Qiu, B.; Jiang, X. A one-step electrochemiluminescence immunosensor preparation for ultrasensitive detection of carbohydrate antigen 19-9 based on multi-functionalized graphene oxide. *Biosens. Bioelectron.* **2015**, *66*, 468-473.
